# Texturing Fermented Emulsion Gels from Soy Protein: Influence of the Emulsifying Agent—Soy Protein vs. Pectin Microgels—On Gel Microstructure, Rheology and Tribology

**DOI:** 10.3390/foods11030294

**Published:** 2022-01-22

**Authors:** Gabriela Itziar Saavedra Isusi, Domenica Paz Puga, Ulrike Sabine van der Schaaf

**Affiliations:** Chair of Food Process Engineering, Institute of Process Engineering in Life Sciences, Karlsruhe Institute of Technology, Gotthard-Franz-Str. 3, Building No. 50.31, D-76131 Karlsruhe, Germany; gabriela.saavedra@kit.edu (G.I.S.I.); domenica.puga@student.kit.edu (D.P.P.)

**Keywords:** soy protein gel, microgel particles, emulsions, tribology, texture

## Abstract

Soy-based yoghurt alternatives are nowadays preferred by consumers. However, they are often perceived as too firm or too soft, sandy, or fibrous. In order to improve this, fibres, especially as in form of microgel particles (MGP), and fats are added to the soy matrix to create a creamy mouthfeel. Both fat and pectin-based MGP can interact with each other and with the protein matrix, creating different microstructures. This can influence the rheological and tribological properties of plant-based protein gels. This works focuses on the effect droplet stabilisation (coconut oil) on the rheological and tribological behaviour of the fermented stirred soy protein gels. For this, fat droplets were stabilised with MGP, SPI, or a mixture of both. Whilst the rheological behaviour remained unchanged for all investigated samples, the tribology of the samples depended on the emulsifier used. The addition of fat decreased the traction coefficient compared to the reference samples without fat. Even though all samples had the same fat content and identical droplet sizes, differences were observed in their lubricating properties. Droplets stabilised solely with SPI presented the best lubricating properties, as indicated by the lowest traction coefficient. Samples stabilised with MGP (or in mixture with SPI) caused higher friction.

## 1. Introduction

Fermented dairy-products are widely consumed all over the world. However, in the last decade, consumers are interested in vegan, vegetarian, or dairy-free lifestyles due to dairy intolerances, environmental concerns, or animal well-being. Soy-based products offer an alternative due to soybeans’ beneficial health effects [1] and techno-functional properties. Nevertheless, consumers are not willing to compromise on the sensory and nutritional properties that are typical for fermented dairy products. Similar to yoghurt, soy-based alternatives are usually fermented with the help of starter cultures [2,3].

During fermentation, the bacteria gradually produce acids, which leads to a drop in the pH value to values between 4.5 and 5.0 [4]. When the pH value reaches these values, the proteins agglomerate due to the lack of electrostatic repulsion between the proteins (glycinin and β-conglycinin) [5]. The result is a product with a hard consistency or texture that lacks creaminess, which is not what consumers demand of yoghurt alternatives [6,7,8]. However, the production of plant-based products is not just about replacing animal protein with plant protein, but also to ensure that vegan alternatives have a similar texture and sensory properties as animal products [9]. Therefore, the achievement of a texture, viscosity, organoleptic, and sensory perception comparable to animal milk-based yoghurt is the main technological challenge in the preparation of soy yoghurt.

The focus of research and development of soy-based dairy alternatives focuses on the improvement of mouthfeel in order to increase the creamy perception of these products [9,10,11]. According to literature [12,13], the creaminess of a product is related to the presence of fat and thickeners, and is linked to its viscosity and lubricating properties. The latter are usually measured by means of oral tribology. Oral tribology is an attractive tool that has been used in the last years by researchers to measure the lubricating properties of soft foods, which correlate to some sensory attributes, such as creaminess [14]. Whilst being consumed, food is being masticated, transported, and swallowed. These processing steps are dominated by friction; thus, depending on whether the consumed food is a good lubricating agent or not, different mouthfeels can be perceived [15,16,17,18]. Various parameters, such as fat content, particle size, and particle deformability, affect the lubrication properties of food [15,16,19].

Therefore, common ways to optimise the textural (rheological and tribological) properties of fermented soy-based products is to add dietary fibre as a thickening agent [20,21,22]. Dietary fibre, such as pectin, has been shown to have positive nutritional effects [20,23] and is highly accepted by consumers. Pectins are often obtained from by-products of the sugar and juice industries and contribute to a circular, sustainable food production. Nevertheless, the creation of a creamy mouthfeel by adding dietary fibre is not trivial [24]: dietary fibre does not fulfil the same functionality as fat globules in a yoghurt network, nor does it possess the globular, particulate shape of the casein micelles that compose a conventional yoghurt [20,25]. The best results, i.e., modulation of creaminess in yoghurt, were obtained by using particulate, deformable soluble fibre [20].

For this reason, the use of microgel particles (MGP) from soluble fibre, such as pectin, could be a promising approach to improve the lubrication properties of fermented soy-based gels. Pectin has been shown to produce stable microgel particles that are able to stabilise emulsions and texturise them [26]. For this reason, pectin-based MGP could be a promising alternative ingredient to modulate the texture of plant-based yoghurts, as it functions as both texturing and emulsifying agent. Nonetheless, as stated by Dagget et al. [13], thickening agents alone are not sufficient to create a creamy mouthfeel. Fat has a lubricating effect that correlates with a creamy mouthfeel [18]. For this, the stabilisation of droplets of a certain size in the protein matrix is required. Whether the fat globules improve (active filler) or worsen (inactive filler) gel properties depends on the emulsifying agent used to incorporate the fat into the protein gel [27]. In this work, we investigated droplets stabilised either with MGP, SPI, or a mixture of both (all-in process). This could translate into different textural impressions when consumed.

The main focus of this work is how the use of either MGP, SPI, or both as emulsifying agents can result in different microstructures that affect the soy gel properties, even though the component quantities remain constant for each sample. Our main hypothesis is that the MGP alone and MGP-stabilised droplets can act as inactive fillers and worsen gel properties. Contrary to this, SPI-stabilised droplets would be incorporated as active fillers. As for droplets stabilised with both MGP and SPI, it is assumed that the interactions between droplets and protein matrix depend on the stabilisation of the individual droplets.

On the one hand, pectin-based MGP build a steric barrier around the droplets [28]. This could prevent any oil–protein interaction due to steric hindrance. Consequently, interactions between droplets and surrounding matrix will only occur in the form of pectin–SPI interactions as only pectin-based MGP will be in contact with the SPI matrix. The literature suggests that ionic hydrocolloids, such as pectin, enhance viscosity, yield stress, and gel firmness from concentrations equal to 0.05% and upwards [22]. On the contrary, neutral hydrocolloids worsen the above-mentioned gel properties. However, it is still unclear as to whether this also applies for pectin-based MGP, as pectin’s charge is decreased due to the MGP formation. Therefore, droplets stabilised by pectin-based MGP might act as either active or inactive fillers. The knowledge on how MGP and fat interact with each other and with the protein matrix is still lacking.

On the other hand, droplets stabilised with SPI are assumed to act as active fillers. Active fillers are usually stabilised by the same protein as found in the continuous phase. The proteins at the interface interact with the surrounding protein molecules, causing an increase in the storage modulus G’ of the protein gel [29]. The effect of active fillers on the protein matrix depends on the concentration of the droplets and the protein content of the continuous phase. When the protein content of the gel matrix is above the gelation threshold, the effect of the active fillers is only limited [30]. Gu et al. (2009) [31] successfully produced SPI-based emulsion-filled gels and demonstrated that the addition of fat (palm-stearin) resulted in harder gels. However, their gels contained 8 wt% SPI and 20% fat. Therefore, it is still unknown as to whether this would be the case for the low-fat system. However, when SPI is used as emulsifying agent, pectin-based MGP are still part of the formulation. The remaining MGP in the protein matrix can also interact with the protein matrix by acting as lubricating agents and possibly worsening the texture of the soy yoghurts. The effect of MGP and fat globules could add up and cause sensorial properties to change in an unknown manner. Therefore, a systematic investigation on this matter is necessary.

Lastly, when both SPI and MGP are used as emulsifying agents, SPI and MGP can also compete with each other and displace each other from the interface, thus affecting the soy gel properties in an unknown manner. For this reason, this works focuses on the effect of fat (coconut oil) and of pectin-based MGP on the rheological and tribological behaviour of the fermented stirred soy protein gels.

This work aimed to show that even though the amount of each component is kept constant, the microstructure that could be obtained is reflected in the rheological and tribologcial properties of the soy protein gel. Hence, one could modulate the sensorial properties of fermented soy protein gels by choosing an emulsifying agent.

## 2. Materials and Methods

### 2.1. Materials

Low methyl-esterified sugar beet pectin (SBP) was gifted by Herbstreith and Fox (Neuenbürg, Germany). The pectin had a degree of esterification of 39%, a degree of acetylation of 5%, and a galacturonic acid content of 65%, according to the supplier’s specifications. Calcium chloride di-hydrate was obtained from Merck KGaA (Darmstadt, Germany). Soy protein isolate (SPI) was kindly provided by Danisco Deutschland GmbH. Starter cultures (*Lactobacillus bulgaricus* and *Streptocuccus thermophilus*) were purchased from Metafood GmbH (Frankfurt, Germany). D-Saccharose was purchased from Carl Roth (Karslruhe, Germany). Coconut oil was obtained from Chemiekontor GmbH (Mannheim, Germany).

### 2.2. Preparations of Pectin Solution

Sugar beet pectin solutions, with pectin mass concentration of 2 wt%, were prepared by dissolving 4 g pectin in 196 g demineralised water in a 600 mL beaker at 60 °C. Pectin was dissolved using a high-shear mixer Ultraturrax T-25 digital (IKA^®^ Werke GmbH and Co. KG, Staufen, Germany) at a rotational speed of 10.000 rpm for 30 s. Afterwards, the solutions were left to cool down to room temperature.

### 2.3. Preparation of Pectin MGP Suspensions

The pectin solutions prepared as described above were used for the preparation of MGP suspensions with a 50 wt% MGP concentration according to the method described by Saavedra Isusi et al. [28]. Gelation was achieved by adding a 40 mM CaCl_2_ solution to the pectin solution under constant shearing at 13.000 rpm for 1 min.

### 2.4. Preparation of Emulsions

Coconut oil-in-water emulsions were prepared by dispersing coconut oil (disperse phase) into a continouos phase, which contained either MGP, SPI, or a mixture of both in a 1:1 ratio. Each emulsion had a total mass of 150 g. The exact composition of each emulsion, as well as the processing parameters, are found in Table 1.

The concentration of MGP used for emulsion stabilisation was reached by dissolving a MGP suspension, containing 50 wt% MGP, in demineralised water.

Prior to the emulsification process, the coconut oil was melted at 40 °C and the continuous phases were also heated to the same temperature. The coconut oil was dispersed into the continuous phase under constant mixing with a high-shear mixer Ultraturrax T-25 digital (IKA^®^ Werke GmbH and Co. KG, Staufen, Germany) at a rotational speed of 15.000 rpm over 30 s in a 600 mL beaker. Afterwards, the emulsion premixes were dispersed for another minute at the same rotational speed. Fine emulsions were obtained by homogenising the coarse emulsions using a rotor-stator system IKA Magic-LAB^®^ (IKA^®^ Werke GmbH and Co. KG, Staufen, Germany) at different rotational speeds (see Table 1), in order to obtain similar droplet sizes. Each emulsion type was prepared in triplicate if not stated otherwise.

### 2.5. Preparation of Soy Protein Gels

Soy protein gels were prepared by means of fermentation. Three formulations were investigated, which were emulsion-filled gel (sample names: MGP, SPI, and MGP + SPI). The emulsion-filled gels were produced by mixing the previously prepared emulsions with a soy protein solution. The protein solution, besides SPI, also contained MGP if not already employed as emulsifying agents during emulsion preparation. The amount of SPI and/or MGP used for droplet stabilisation was taken into account to reach the final concentration of each component, and therefore they remained constant, and equalled 5 wt% SPI, coconut oil content of 4 wt%, and 1 wt% MGP for all emulsion-filled gels. The preparation method of each emulsion is found in the previous sections. The exact composition of each soy protein solution is found in Table 2. Each sample had a total mass of 500 g (350 g SPI solution with or without MGP + 150 g emulsion). Additionally, a reference sample (fermented SPI gel) was also prepared (500 g). This sample contained neither MGP nor coconut oil.

The soy protein solutions were all prepared in the same manner. SPI powder was dissolved in demineralised water at 60 °C for at least 30 min. Then, the SPI solutions were pasteurised by heating them at 80 °C and holding the temperature for 10 min. Afterwards, the protein solutions were let to cool down to 43 °C. Upon cooling, the 150 g emulsion was added to the SPI solutions for samples MGP, SPI, and MGP + SPI. Then, 1 wt% saccharose and yoghurt cultures were added to all samples. All mixtures were poured into individual commercially available yoghurt cups and sealed with an aluminium foil. Samples were let to ferment in a water bad at 43 °C, until the pH of the samples reached 4.5 ± 0.2. The reference sample was prepared in the same manner as the others. SPI was dissolved in demineralised water at 60 °C for at least 30 min. The SPI solution was then pasteurised at 80 °C for 10 min. After pasteurisation, the solution was cooled down to 43 °C for inoculation. The fermentation was ended when the pH reached a value of 4.5 ± 0.2. After fermentation, the pot-set SPI gels were stirred for 30 s at 3.000 rpm and cooled for at least 24 h at 5 °C prior to analysis. All formulations were prepared in triplicate.

### 2.6. Measurement of Oil Droplet Size Distribution

The droplet size distribution (DSD) of the prepared emulsions was determined by static laser light scattering using a HORIBA LA-950 Particle Analyser (Retsch Technology, Haan, Germany). The results are shown as the cumulative volume distribution Q_3_. The refractive indices were set at n = 1.475 for coconut oil and n = 1.333 for water for all emulsions. The determination of the droplet sizes was made following the Fraunhofer theory. All measurements were conducted in triplicate at room temperature.

All emulsion samples were observed under an Eclipse LV100ND Microscope (Nikon GmbH, Düsseldorf, Germany), equipped with a DS-Fi1c camera and a temperature control stage Linkam LTS 420 (Linkam Scientific Instruments, Tadworth, United Kingdom). Micrographs of the samples were taken with 10- or 20-fold magnification lenses at 5 °C.

All prepared samples were freeze-dried and broken into pieces. The gel pieces were analysed using scanning electron microscopy (SEM) at 50-fold magnification.

### 2.7. Tribological Measurements

The traction coefficient (also referred to as coefficient of friction CoF) of the investigated samples was measured using a stress-controlled HAAKE Mars rheometer (Thermo Electron GmbH, Karlsruhe, Germany), equipped with a ball-on-3-plates TR13 45° tribology measuring geometry. A stainless-steel ball and 3 polydimethylsiloxane (PDMS) plates (Sylgard 184, Dow Chemical, Midland, MI, USA) were used as tribo-pairs. All measurements were conducted at a relative sliding speed range of v_R_ = 1 − 1000 mm s^−1^, where 100 measurement points were taken. The axial force F_N_ was set to 1 N and temperature to 20 °C. Each individual preparation of a sample was analysed at least 5 times.

### 2.8. Rheological Measurements

All rheological analysis were conducted using a stress-controlled Physica MCR 301 rotational Rheometer (Anton Paar, Graz, Austria). The measurements were conducted at 20 °C with a plate–plate geometry PP25, and a 1 mm gap. Prior to each measurement, all samples were given 5 min to equilibrate, thus setting constant measurement conditions.

Amplitude sweeps were performed at an angular frequency of ω = 1 rad s^−1^ over a range of amplitude stresses from 0.1 to 100 Pa. The amplitude stress was increased logarithmically, and 11 measurement points were taken for each decade. All amplitude sweeps were conducted in triplicated for each sample,

Frequency sweeps were done at an amplitude stress of 1 Pa (stress within the linear viscoelastic region of all samples) and over a frequency range from 50 to 1 rad s^−1^. The angular frequency was increased in a logarithmic manner and 22 measurements points were recorded pro decade. Frequency sweeps were conducted in duplicate for each sample.

### 2.9. Statistical Analysis

Each sample preparation was made in triplicate. If not specified otherwise, all analyses were conducted at least three times per independent test. All data were assessed by a multifactorial analysis of variance (ANOVA) and a Tukey test as post hoc test. Dissimilarities in samples were considered statistically relevant at a level of *p* ≤ 0.05. The software OriginPro 2019 (OriginLab Corp., Northampton, MA, USA) was used for the statistical analysis, calculation of averages, and standard deviations.

## 3. Results and Discussion

### 3.1. Droplet Size Distribution

**Figure 1:**
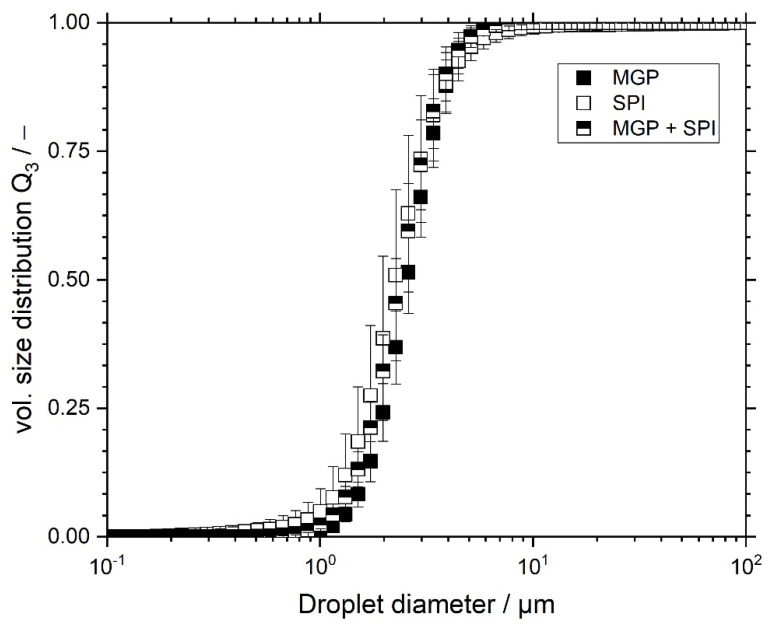
Coconut oil-in-water emulsions (20 wt% oil), stabilised with pectin-based microgel particles (MGP), soy protein isolate (SPI), or both in a ratio of 1:1 (MGP + SPI).

In order to assess the influence of the emulsifying agent and the addition of MGP on the rheological and tribological properties of soy protein gels, one should minimise the effect of the oil droplet size and droplet size distribution. For this reason, we aimed for the droplet sizes of all three emulsions to be in the same diameter range. The obtained droplet size distributions for all emulsions (MGP, SPI, and MGP + SPI) are found in Figure 1.

As seen in Figure 1, all emulsions possessed similar oil droplet distributions, with mean Sauter diameters equal to 2.6 ± 0.1 µm, 2.7 ± 0.1 µm, and 2.5 ± 0.1 µm for emulsions stabilised with MGP, SPI, and MGP + SPI, respectively. All investigated emulsions were not significantly different at a significance level of *p* < 0.05. Therefore, these emulsions were deemed suitable for the further preparation of SPI gels, as the influence of the oil droplet size was expected to be similar or even identical for all investigated samples.

### 3.2. Influence of Droplet Stabilisation Method on the Rheological Properties

**Figure 2:**
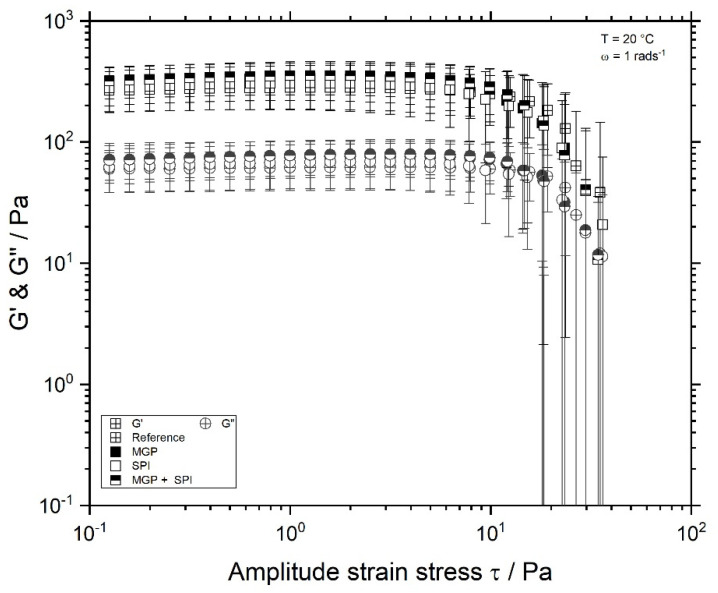
Amplitude test of fermented soy protein isolate (SPI) gels, measured at 20 °C, and an angular frequency of 1 rads^−1^. Reference sample, without oil 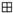 and SPI emulsion gels (droplets stabilised with microgel particles (MGP) 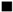, with SPI 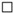, or both MGP + SPI 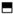).

Fermented SPI gels, prepared with coconut oil droplets stabilised with SPI, MGP, or MGP + SPI as emulsifying agents, were subjected to rheological analysis after a storage period of 24 h at 5 °C. The deformability of the investigated samples was assessed from the linear viscoelastic region (LVE) of SPI gels. Softer materials should sustain greater strain stress than densely packed materials [32]. The maximal stress within the LVE, $\tau_{max}$, is determined by the gel’s strength. An increase of stress strain beyond $\tau_{max}$ leads to changes in the elastic and viscous behaviour of the samples [33]. The effect that the addition of fibre and fat has on the gel properties depends on the type and extent of the interactions between these components and SPI [34]. It was expected that the addition of fibre in form of microgel particles and fat would affect the length of the LVE of the SPI-gels compared to samples without these components [8,20,22]. However, as seen in Figure 2, amplitude tests show no difference between the samples.

All samples exhibited predominantly elastic behaviour, as G′ was greater than G″, and thus the samples can be regarded as solid-like material. This is in in agreement with the results presented by Einhorn-Stoll and Drusch [35]. Moreover, the investigated samples possessed LVE regions of similar length. However, contrary to the expectation, the addition of fat and MGP did not affect the LVE nor $\tau_{max}$ of the samples.

On the one hand, MGP and MGP-stabilised droplets were thought to decrease the gel strength, as pectin-based MGP possesses reduced charge at low pH values [36]. However, the length of the LVE region and the maximal stress strain remained unaffected. The added MGP concentration represented only 1% of the yoghurt, and each MGP was composed of only 2% SBP; thus, pectin represented only 0.02% of the yoghurt mass. This amount is very low compared to the concentrations used in other investigations. The literature suggests that the addition of fibre below 0.1% does not affect the rheological properties of dairy yoghurt [20].

On the other hand, SPI-stabilised droplets were expected to increase the gel strength, as they were assumed to act as active fillers. However, Ningtyas et al. (2021) showed that the effect of oil and/or fat depends both on the type of oil/fat that is incorporated into the soy matrix and on the amount of the disperse phase. Significant changes in gel firmness have been observed for oil concentrations over 10% [31]. Therefore, it can be concluded that the addition of coconut oil at 4% concentration does not affect the gel properties due to the low concentration.

**Figure 3:**
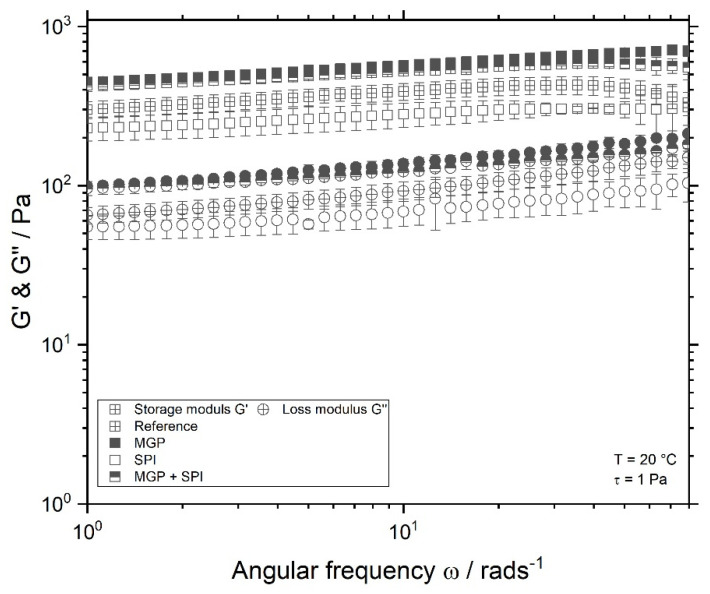
Frequency test of fermented soy protein isolate (SPI) gels, measured at 20 °C, and an amplitude stress strain of 1 Pa. Reference sample, without oil 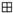 and SPI-emulsion gels (droplets stabilised with microgel particles (MGP) 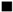, with SPI 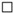, or both MGP + SPI 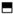).

Additionally to amplitude test, frequency sweeps were conducted. These measurements were performed at small deformations or stress strains, at which the gel structure was not affected (Nöbel 2016 et al.). Figure 3 shows the frequency test of the investigated samples.

As seen in Figure 3, samples containing droplets stabilised with MGP alone or with both MGP and SPI possessed higher G’ values than the other two samples. The storage modulus G’ is dependent on the cross-linking density of gels [37,38]. Higher G’ values indicate stronger gels. Therefore, it can be concluded that MGP and MGP + SPI samples result in denser cross-linked gels. It is assumed, that the oil droplets are covered with MGP. These can act as anchors in the gel matrix [20], thus forming more crosslinks in the protein matrix. Contrary to this, the oil droplets stabilised with SPI act as defects on the protein gel, which causes a minimal decrease in G’, compared to the reference sample. Thus, frequency sweep data show that the type of droplet stabilisation does affect the gel strength.

### 3.3. Flow Behaviour

**Figure 4:**
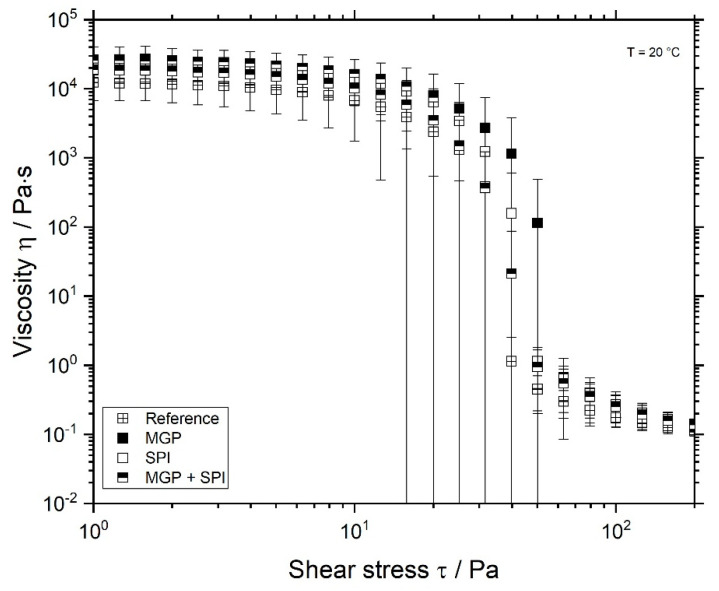
Viscosity of fermented soy protein isolate (SPI) gels over the shear stress, measured at 20 °C. Reference sample, without oil 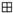 and SPI-emulsion gels (droplets stabilised with microgel particles (MGP) 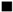, with SPI 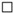, or both MGP + SPI 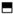).

The viscosity of the prepared SPI gels is depicted in Figure 4. All samples possess a shear-thinning behaviour, which is in good agreement with the investigation conducted by Ningtyas et al. (2021). Here again, no significant difference was observed, even in comparison to the reference sample. These results are in good agreement with those depicted in Figure 2.

The yield stress of all samples was found at around 20 Pa. After exceeding this stress value, all samples began to flow. At this stress, there was also no significant difference among the measured viscosity of all formulations.

### 3.4. Microstructure of Fermented SPI Gels

**Figure 5:**
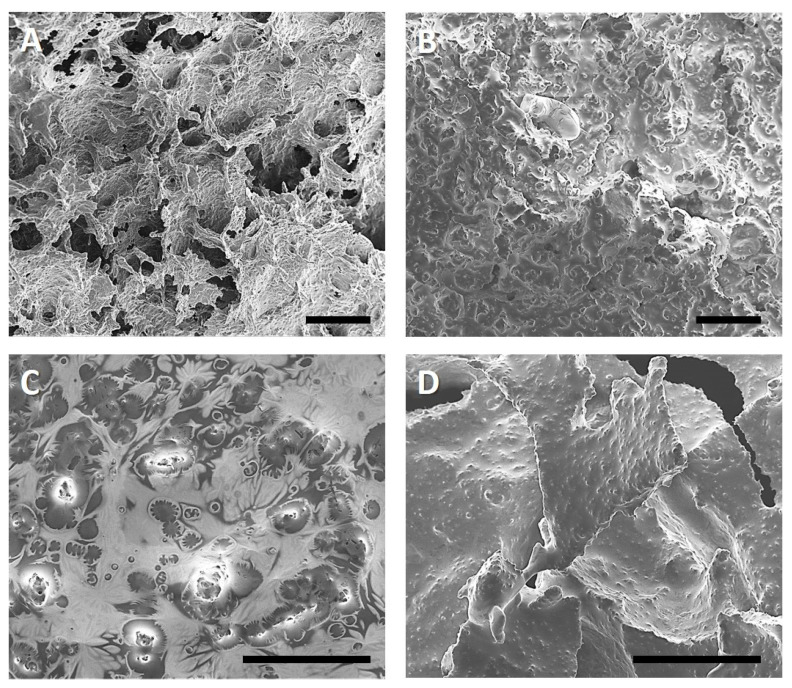
Micrograph obtained using a scanning electron microscope (SEM) of fermented SPI gels after freeze-drying. (**A**) Reference sample, without coconut oil. (**B**) SPI emulsion gel (droplets stabilised with MGP). (**C**) SPI emulsion gel (droplets stabilised with SPI). (**D**) SPI emulsion gel (droplets stabilised with MGP + SPI). Scale bar equals 100 µm.

As the rheological analysis did not show any significant differences amongst the emulsion gels and between them and the reference samples, all prepared gels were freeze-dried in order to observe them using a scanning electron microscope. The aim of this was to assess whether the emulsion gels and the reference samples (MGP-free and fat-free) possessed the same structure. The obtained micrographs are depicted in Figure 5. The scale bar equals 100 µm.

**Figure 6:**
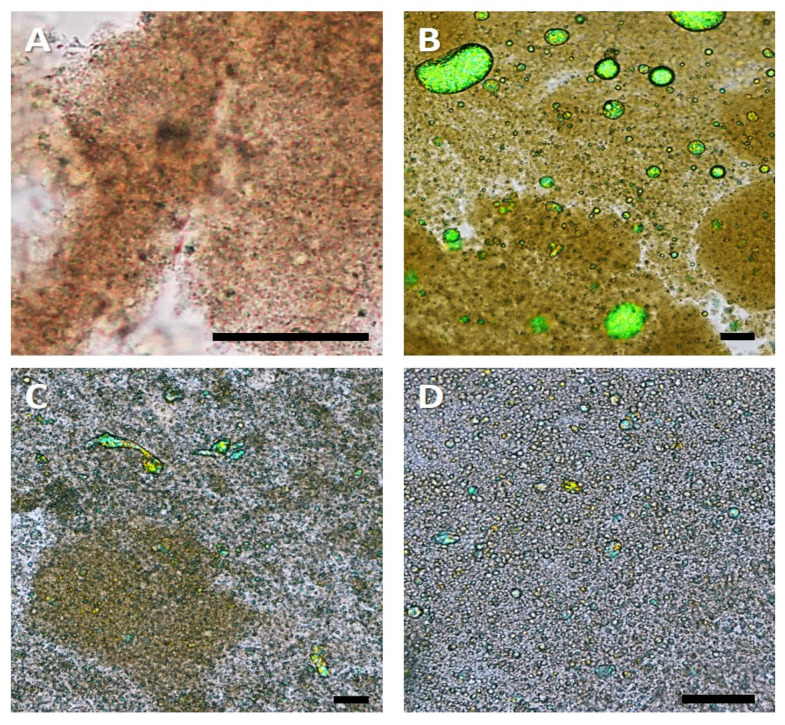
Micrograph obtained using light microscopy and a polarisation filter of fermented SPI gels. (**A**) Reference sample, without coconut oil. (**B**) SPI emulsion gel (MGP). (**C**) SPI emulsion gel (SPI). (**D**) SPI emulsion gel (MGP + SPI). Scale bar equals 50 µm.

As seen from Figure 5, there were notable structural differences between all prepared samples. Figure 5A (upper left) shows the reference sample. It can be observed that this gel lacked oil droplets and MGP. Additionally, it is noticeable that the gel network was composed of coagulated proteins, possessing larger pores in which water was entrapped prior to the freeze-drying process. Figure 5B displays an SPI emulsion gel, wherein the oil droplets were stabilised using solely pectin-based MGP (formulation MGP). This gel presents many bumps that are assumed to be MGP-covered fat droplets. These appear to be larger than the droplets measured by laser light scattering (Figure 1). This could be caused by coalescence, as their diameter is larger than the initial droplet diameter. The cause of droplet coalescence could be attributed to the freeze-drying process, or the gelation process. Another explanation could be the agglomeration of MGP at the interface, which could appear as larger particles or droplets. Nevertheless, this cannot be concluded without a doubt from the presented micrograph. Therefore, further micrographs were taken using a heat-cooling stage. The obtained images are discussed further below (Figure 6).

Figure 5C shows a SPI emulsion gel, in which droplets were stabilised using SPI alone (formulation SPI). Here, MGP were added separately, and thus they were expected to remain in the protein matrix and they should not have adsorbed onto the droplet interface. This can indeed be recognised in Figure 5C. The darker spots depicted in the image are fat droplets, incorporated into the SPI matrix. Moreover, MGP can be observed in the protein matrix. In this case, they appeared to be single MGP agglomerates. Figure 5D depicts a SPI emulsion gel whose droplets were stabilised with SPI and MGP at the same time (MGP + SPI formulation). Compared to the other two emulsion gels (Figure 5B,C), the structure of this formulation appeared to be more homogeneous. Here, the fat droplets were much smaller than the ones depicted in Figure 5B. This supports the assumption that droplets in emulsion gel MGP underwent some kind of coalescence or agglomeration. Additionally, the structure of MGP + SPI formulation displayed smaller “bumps”, which were assumed to be MGP-covered droplets. Previous work of ours [39] has shown that MGP agglomeration is hindered in the presence of oil droplets. This could explain why the MGP shown in Figure 5D appeared smaller than in the other two formulations.

The differentiation between MGP and droplets from the shown micrographs is not possible without doubt. For this reason, light microscopy using a heat-cooling stage and a polarisation filter was performed. Due to the polarisation filter, in combination with an analyser plate and a lambda-plate, crystalline structures displayed colours in the images. With this technique, one can differentiate between MGP and coconut fat (crystalline). The obtained images are found in Figure 6.

Figure 6A shows the reference sample, which did not contain oil. Therefore, the micrograph lacked green shimmering droplets. As seen from Figure 6B–D, there were some differences in the droplet size of the emulsion gels. Figure 6B displays the fermented SPI emulsion gel, stabilised with MGP. It is noticeable that the fat droplets were larger than the initial mean Sauter diameter of the emulsion prior to the fermentation. Since these samples were not freeze-dried, the cause of droplet coalescence cannot have been in the drying process. Hence, the assumption that the droplets coalesce during the fermentation process is validated.

Figure 6C,D displays smaller droplets. However, these did not have exactly the same size as the ones in the initial emulsions. Therefore, it can be assumed that, here as well, some degree of coalescence took place during the fermentation process or the cooling process. Nevertheless, this was not comparable to the extent of size increase of the sample stabilised solely with MGP. Figure 6C shows some protein–droplet agglomerates. This could be caused by the interactions between the proteins in the continuous phase and the proteins stabilising the droplet’s interface [40]. Figure 6D shows very evenly distributed droplets in the protein matrix. This somewhat agrees with the SEM micrographs, depicting the most homogenous structure. The sample depicted in Figure 6D (MGP + SPI) was stabilised with both MGP and SPI. This translated into a lower concentration of SPI adsorbed onto the interface, which could hinder protein–protein interaction between droplets.

As seen from the micrographs discussed in this section, the method by which droplets were stabilised caused changes in the microstructure of the fermented SPI gels. Although these arrangements did not translate into difference in the rheological behaviour, they might have had an impact on the surface or lubricating properties of the samples. Therefore, tribological measurements were conducted.

### 3.5. Tribology of Fermented SPI Gels

**Figure 7:**
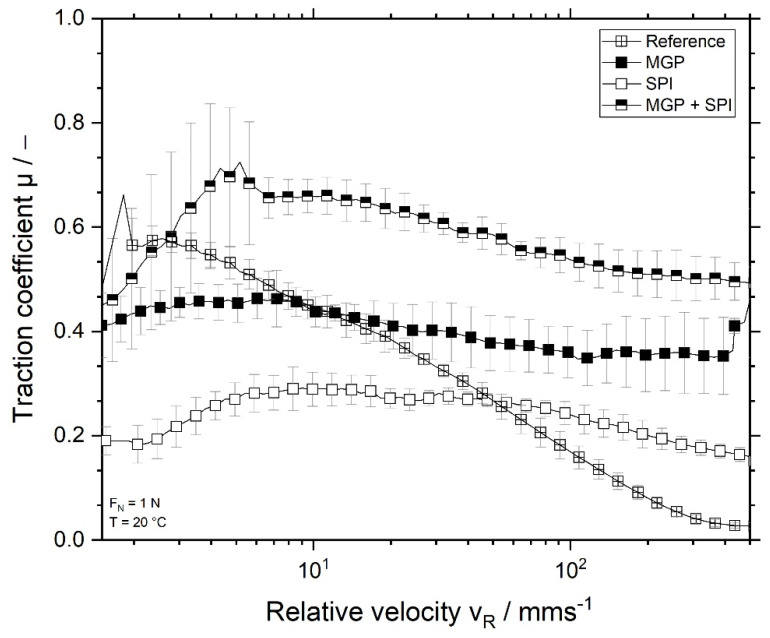
Traction coefficient of fermented SPI gels over the relative (sliding) velocity, measured at 20 °C, using PDMS pins and a stainless steel ball as tribo-pairs. Reference sample, without oil 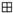 and SPI-emulsion gels (droplets stabilised with MGP 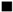, with SPI 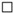, or both MGP + SPI 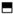).

Several authors have demonstrated that the creamy perceptions of a semi-solid product correlates with the friction these products cause upon consumption [16,18]. Therefore, tribology can provide an insight into the mouthfeel of the investigated samples. The lubricating properties of fermented SPI gels were determined at 20 °C, and the results are depicted as Stribeck curves of the samples (Figure 7).

As seen from Figure 7, the samples showed different lubricating properties. The reference sample had the highest traction coefficient of all SPI gels at low relative velocities. As the sliding speed increased, the samples began to spread and lubricate the tribo-pairs, thus decreasing the friction between the surfaces. This behaviour is typical of protein, as they act as good lubricants [41,42]. Samples containing oil possess lower friction coefficients at lower relative velocities. This could be attributed to the presence of oil [16,43]. Wijk and Prinz (2005) demonstrated that the droplet size and concentration affected the friction caused by the semi-solid products, and consequently influenced their creamy perception: small droplets and high fat concentration decreased friction and resulted in a creamy perception. Nevertheless, all samples investigated in this work possessed the same amount of fat droplets of nearly identical sizes. However, even though all SPI emulsion gels contained the same amount of oil, they lubricated in different manners. Whereas the sample stabilised solely with SPI had the best lubrication, as indicated by the lowest traction coefficient, the samples stabilised with MGP (both in mixture with SPI and alone) caused higher friction. This could have been due to the presence of MGP at the interface. As MGP builds a steric protective layer around the droplets, this could make the droplets more resistant towards the stress applied onto them. It has been proven that Pickering and Pickering-like emulsions are more resistant towards stress than droplets stabilised with protein or other surfactants [44]. This can prevent the oil from spreading and covering the tribo-pairs, which can result in a less lubricating effect [16]. The sample stabilised with both MGP + SPI has the highest traction coefficient of the SPI emulsion gels. As seen from the micrographs, the MGP + SPI sample had a more homogenous structure, with evenly dispersed droplets and MGP, which were of smaller size. The presence of these particles could increase the friction, as proven by other authors for dairy products [45]. Additionally, Engelen et al. [17] showed that the presence of (solid) particles can even counteract the lubricating properties of fat. This could also apply for pectin-based MGP.

Moreover, the samples containing oil did not decrease friction further as the relative speed increases, as was the case for the reference sample. Hence, no transition to the mixed lubrication regime was observed for these samples. The reference sample showed a “stick and slide” pattern [46]. This kind of behaviour is typical for low-fat products: at low relative speeds, the reference sample forms a thin lubricating film. For this reason, the friction depends mainly on the roughness of the tribo-pair. As the speed increases, the reference sample flows into the contact zone between the tribo-pairs, thus separating them and reducing friction (mixed regime) [46]. Fat droplets extend the boundary regime to higher speeds. It is suggested that this extension is caused by the coalescence of fat globules during the measurement. Fat droplets coalesce and form a (thicker) lubricating film, which can reduce the friction between the tribo-pairs, without having to separated them (transition to mixed and hydrodynamic regimes) [46,47]. Thus, the lubrication properties of samples containing coconut oil is dominated by the fat and not by the proteins.

## 4. Conclusions

Soy-based dairy alternatives do not form gels like conventional dairy products do. Moreover, most of these soy alternatives lack fat. Due to these differences, the sensorial properties of these products are not comparable to the dairy ones. In order to improve this, fibres, alternatively in the form of microgel particles (MGP), and fats might be added to the soy matrix to create a creamy mouthfeel. However, it still remains unclear as to how the presence of both fat and pectin-based MGP can influence the rheological and tribological properties of plant-based protein gels. In this work, the effect of fat (coconut oil) and of pectin-based microgel particles on the rheological and tribological behaviour of fermented stirred soy protein gels was investigated. MGP, soy protein, or a mixture of both was used as emulsifying agents in order to embed the coconut oil droplets into the soy protein matrix. Even though the soy protein gels had the same formulation, different microstructures were obtained by varying the stabilisation process. These microstructures were thought to affect the rheological and tribological behaviour of the soy gels. By doing so, the mouthfeel of the soy gels is expected to be affected as well.

The rheological behaviour remained unchanged for all investigated samples, despite of the presence or lack of fat. However, this was not the case for the tribology of the samples. The tribology of the samples depended highly on how the oil droplets were stabilised. As expected, all SPI emulsion gels possessed a lower traction coefficient compared to the reference samples without added fat. However, even though all samples had the same fat content and droplets in nearly identical sizes, noticeable differences were observed in their lubricating properties. Droplets stabilised solely with SPI presented the best lubricating properties, as indicated by the lowest traction coefficient. However, whether the measured differences in lubricating properties also translate into different sensorial properties of the gels remains unknown. In order to confirm that these differences are also perceived upon consumption, we require a correlation between tribology and a sensorial panel. Nevertheless, this study shows that the lubricating properties and the microstructure of a constant formulation can be tuned by the preparation process, i.e., the method used for droplet stabilisation.

## Figures and Tables

**Table 1:** Composition of coconut oil-in-water emulsions. MGP refers to microgel particles, SPI refers to soy protein isolate.

Sample Name	Oil Content (g)	MGP Concentration (g)	SPI Content (g)	Water Content (g)	Rotational Speed (rpm)
MGP	20	5	0	125	26,000
SPI	20	0	5	125	15,000
MGP + SPI	20	2.5	2.5	125	25,000

**Table 2:** Composition of investigated yoghurts.

Sample Name	SPI Content (g)	MGP Content (g)	Water Content (g)	Emulsion Content (g)
Reference	25	0	475	0
MGP	25	0	325	150
SPI	22.5	5	322.5	150
MGP + SPI	22.5	2.5	325	150

## Data Availability

Data available on request.
